# Applications of Antibody-Based Antigen Delivery Targeted to Dendritic Cells In Vivo

**DOI:** 10.3390/antib11010008

**Published:** 2022-01-25

**Authors:** Jessica Bourque, Daniel Hawiger

**Affiliations:** Doisy Research Center, Department of Molecular Microbiology and Immunology, School of Medicine, Saint Louis University, 1100 S. Grand Blvd., St. Louis, MO 63104, USA; jess.bourque@health.slu.edu

**Keywords:** dendritic cells, antigen targeting, antigen delivery, chimeric antibodies, autoimmunity, immunity, tolerance, immunizations

## Abstract

Recombinant immunoglobulins, derived from monoclonal antibodies recognizing the defined surface epitopes expressed on dendritic cells, have been employed for the past two decades to deliver antigens to dendritic cells in vivo, serving as critical tools for the investigation of the corresponding T cell responses. These approaches originated with the development of the recombinant chimeric antibody against a multilectin receptor, DEC-205, which is present on subsets of murine and human conventional dendritic cells. Following the widespread application of antigen targeting through DEC-205, similar approaches then utilized other epitopes as entry points for antigens delivered by specific antibodies to multiple types of dendritic cells. Overall, these antigen-delivery methodologies helped to reveal the mechanisms underlying tolerogenic and immunogenic T cell responses orchestrated by dendritic cells. Here, we discuss the relevant experimental strategies as well as their future perspectives, including their translational relevance.

## 1. Introduction

**Figure 1:**
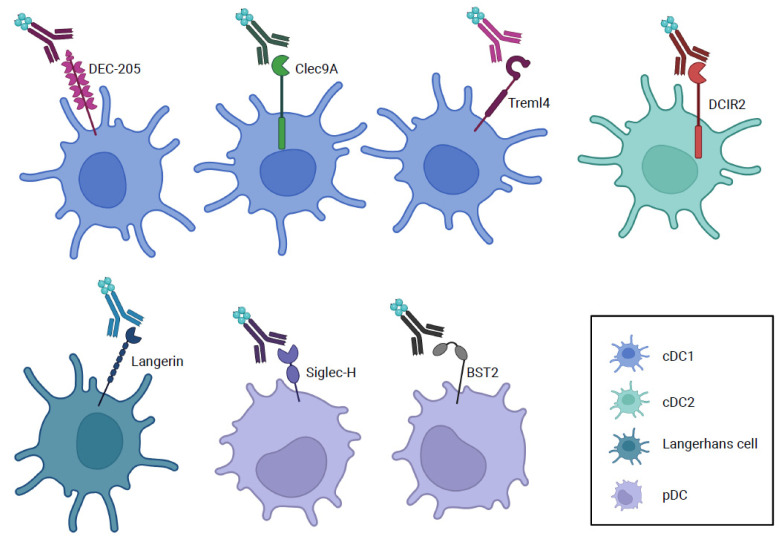
Various receptors used for targeted antigen delivery to dendritic cells. cDC: conventional dendritic cell; pDC: plasmacytoid dendritic cell.

Dendritic cells (DC) take up, process, and present foreign and self-antigens to T cells, resulting in critical interactions that initiate and regulate specific immune responses. DC consist of two main populations, plasmacytoid (pDC) and conventional (cDC), which are further divided into the cDC1 and cDC2 subsets based on their developmental dependency on IRF8/Batf3 and IRF4/Notch2 transcription factors, respectively, and also their specific expression of multiple surface markers [1,2,3,4,5,6]. Whereas pDC express DC-SIGN, Siglec-H, B220, and Ly6c, murine cDC1 are characterized by specific expression of XCR1, and some cDC1 also express CD8α, DEC-205, BTLA, Langerin, Treml4, CD103, and Clec9a. In contrast, murine cDC2 express CD172a (SIRPα) and additionally DCIR2 and CD11b [3,4,7,8,9]. Many of these surface markers represent molecules that had been utilized for antigen-delivery strategies to the corresponding cDC, the primary focus of this review, and pDC, as discussed below (Figure 1).

Both human and murine DC develop from bone marrow-derived progenitors that disseminate throughout multiple immune and non-immune tissues and organs and have distinct phenotypic characteristics, as reviewed in [5,6,10]. In contrast to cDC, which are present at anatomical barriers such as the skin or intestines, and the associated lymphoid tissues that are exposed to microbiota, other cDC, referred to here as “systemic” cDC, are present in secondary lymphoid organs, including the spleen and lymph nodes (LNs), and are not directly exposed to extrinsic environmental factors. Instead, these cDC constantly survey local and circulating antigens derived from parenchymal, interstitial, and other non-barrier tissues. Further, systemic cDC also present to T cells other relevant antigens, notably those derived from infectious pathogens as well as the antigens included in vaccines [11,12,13]. The cDC that are present in lymphoid organs either originate from the progenitors directly disseminating through the blood or from progenitors that first seed peripheral non-lymphoid tissues and then fully differentiate into cDC before migrating to lymphoid organs [6,14]. Such migratory cDC may ferry antigens from either healthy tissues or tumors and present these antigens to T cells directly, or instead, pass on the antigens to other cDC residing in the lymphoid tissues [11,15,16,17,18,19,20,21,22,23,24,25,26,27,28]. Overall, in contrast to the antigens that can become readily available at the anatomical barriers, the experimental administration of the antigens to systemic cDC has historically involved injections of substantial amounts of the relevant free peptides or proteins, often leading to non-specific effects [9,29]. Therefore, the development of antigen-delivery systems based on recombinant antibodies specific for cDC opened the door to precise and selective methods of antigen delivery and allowed for a rigorous analysis of the resulting impact on T cells. Additionally, a combination of the antigen-delivery methods with genetic models that alter the numbers or specific functions of cDC further broadened our understanding of the roles of various cDC populations [9]. Moreover, the methods of targeted antigen delivery to cDC became promising avenues for novel immunotherapeutic applications [30,31,32,33,34].

Antigens can be delivered to various subsets of dendritic cells in vivo using recombinant chimeric antibodies to specific receptors that are present on such subsets. 

## 2. Anti-DEC-205—The First Recombinant Chimeric Antibody for In Vivo Antigen Delivery

**Figure 2:**
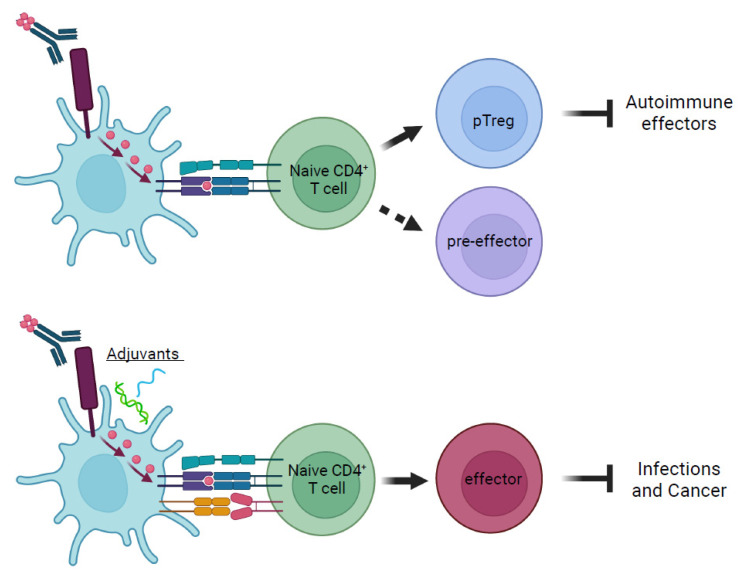
T cell outcomes of targeted antigen delivery to DC in the absence and presence of pro-inflammatory signals.

The first recombinant chimeric antibody ever designed to deliver specifically defined T cell antigens to DC targeted the endocytic receptor DEC-205 (DEC205, CD205, LY75). DEC-205 is expressed at high levels on cDC1 in the steady state, defined as the absence of specific pro-inflammatory stimuli [11,35,36,37]. Under pro-inflammatory conditions, such as in vivo after immunization using adjuvants or in vitro after specific activation, other immune cells, including germinal center B cells and cDC2, can also increase expression of DEC-205 [29,38,39,40]. However, in the steady state, DEC-205 mediates an efficient endocytic pathway, allowing for the robust processing and presentation of antigens specifically delivered to DEC-205^+^ cDC1 [29,32,41,42]. Further, antigens delivered through DEC-205 are presented both in the context of major histocompatibility complex class I (MHC-I) and MHC-II without causing other perceivable changes to cDC [32,35,43]. Therefore, DEC-205 has become an ideal target for antigen-delivery strategies to cDC1 in the steady state and has allowed for a comprehensive analysis of the outcomes of T cell activation by cognate antigens presented by cDC [32,44,45,46,47] (Figure 2).

These early experiments helped to unequivocally establish that, in the steady state, the outcome of the initial CD4^+^ T cell antigenic stimulation by DC results in T cell tolerance [35]. By clarifying the functions of cDC in the steady state, these results helped to refute the original designation of such cDC as “immature” immune bystanders, as was initially assumed due to an apparent lack of T cell priming in the absence of pro-inflammatory stimuli [9,29,35,48]. Furthermore, by using MHC-I-restricted and cross-presented antigens, subsequent experiments extended the initially established tolerogenic functions of the DEC-205^+^ DCs to the induction of tolerance among CD8^+^ T cells [43]. Such tolerogenic functions of cDC also represent potential important therapeutic opportunities, and anti-DEC-205-mediated delivery to cDC1 was subsequently harnessed for the prevention and mitigation of an autoimmune process. In these experiments, animals were treated with anti-DEC-205-myelin oligodendrocyte glycoprotein (MOG_35-55_) chimeric antibody (designated as anti-DEC-205-MOG or anti-DEC-MOG) to deliver the neural antigen MOG to cDC1 under non-inflammatory conditions, resulting in a prevention of specific autoimmune responses and disease symptoms of experimental autoimmune encephalomyelitis (EAE), a murine model of multiple sclerosis (MS) [36]. The effectiveness of targeting antigens to DEC-205 to promote antigen-specific immune tolerance and to ameliorate disease severity was subsequently extended to other models of autoimmune disease, including diabetes, inflammatory bowel disease (IBD), graft-versus-host disease (GVHD), and arthritis [49,50,51,52,53,54] (Table 1).

Whereas in the absence of pro-inflammatory signals (steady state) presentation of antigens by cDC to T cells results in the formation of pTreg cells and also some other T cell types, such as pre-effectors, antigenic presentation in the presence of pro-inflammatory adjuvants leads to effector T cell responses.

In contrast to these tolerogenic mechanisms, the efficient priming of effector T cells by cDC in vivo requires sensing of additional signals, such as those relayed through specific pattern recognition receptors that recognize conserved pathogen components [6,67,68,69,70,71]. These signals result in a pro-immunogenic process that enhances specific functions of cDC and is generally referred to as “maturation”, which results in altered expression of costimulatory molecules, MHCs, and cytokines, as well as other changes in cDC to facilitate enhanced effector T cell-priming under pro-inflammatory conditions [69,72,73]. Consistently, the activation of cDC1 through ligation of surface CD40 prevented a tolerogenic outcome of T cell activation and resulted in the priming of antigen-specific effector T cells [35]. Further, the targeting of antigens to DEC-205 has also been successfully applied as a promising vaccine approach in both infectious and tumor models. Administration of the adjuvant polyinosinic:polycytidylic acid (Poly(I:C)) together with recombinant anti-DEC-205 antibodies linked with the Yersinia pestis virulence protein LcrV induced humoral responses as well as T helper 1 (Th1) CD4^+^ T cells in the lung [63,64]. Similarly, administration of anti-DEC-205-HIVgag-p24 in the presence of an anti-CD40 adjuvant led to Th1 responses as well as cross-presentation to CD8^+^ T cells [74]. In addition to eliciting immune responses against pathogens, the delivery of tumor-associated antigens (such as tyrosinase-related protein 2) or other antigens known to be involved in tumorigenesis (such as survivin) to DEC-205-expressing cells in the presence of co-administered adjuvants, such as CpG, Poly(I:C), or anti-CD40, enhanced the anti-tumor CD4^+^ and CD8^+^ T cell responses and decreased the tumor burden [57,58,59,60,61,75,76].

## 3. Orchestrating Diverse Immune Responses by Delivering Antigens to Dendritic Cells in the Steady State

The results of experiments that revealed priming of immune responses, resulting from combining antigen delivery to DEC-205 with the administration of adjuvants, reinforced the dogma of tolerogenic responses being induced in the steady state while immune responses were promoted under pro-inflammatory conditions [9,29]. However, it is important to note that the robust induction of tolerance by cDC in the steady state is not consistent with other experimental evidence that indicated an induction of effector T cells and autoimmunity, even in the absence of major pro-inflammatory perturbations in homeostasis [77,78,79,80,81,82,83,84,85,86]. Further, the crucial process of cancer immunosurveillance postulated to constantly remove cancerous cells arising under homeostatic conditions may be viewed as inconsistent with the predominantly tolerogenic outcomes of T cell activation mediated by cDC1 under homeostatic conditions [87,88]. Recent experiments utilizing anti-DEC-205-mediated antigen delivery in the steady state have helped to clarify these conundrums. These results identified an active programming in the steady state by cDC1 of naïve CD4^+^ T cells with specific epigenetic and transcriptional instructions, leading to an acquisition of T helper effector functions (Figure 2). Such “pre-effectors” activated in the steady state become poised for subsequent effector differentiation and, upon re-stimulation under non-skewing conditions in vitro or in vivo, readily express key factors such as IFN-γ that then can trigger expression of T-bet, and possibly other effector master regulators [89]. These pre-effectors can contribute to initiation of the autoimmune responses, and any such pre-effectors specifically induced by tumor neo-antigens might also have a key role for cancer immunosurveillance [89,90].

Such an induction of pre-effectors in the steady state necessitates the existence of dedicated pathways in cDC to promote specific mechanisms of tolerance that include inducing T cell anergy, T cell deletion, and a conversion of peripheral regulatory T cells (pTregs) [9,11,91]. Particularly, the pTregs that are de novo converted in response to specific self-antigens bestow a dominant and long-lasting tolerance to peripheral antigens that can ameliorate various autoimmune responses, therefore crucially complementing the immunological tolerance first initiated in the thymus [9,11,92]. Multiple cytokines, metabolites, and other key molecules, including transforming growth factor β (TGF-β), retinoic acid (RA), IL-10, and CD39, help to initiate and further facilitate the conversion of pTregs [15,93,94,95,96,97,98,99,100,101,102]. Such induction of systemic pTregs is efficiently mediated in the steady state by the Batf3-dependent cDC1, corresponding to previously defined CD8α^+^DEC-205^+^ cDC present both in LNs and spleen [1,9,11,29,47]. These tolerogenic cDC1 are further distinguished by their high expression of B and T lymphocyte associated/attenuator (BTLA), and such BTLA^hi^ cDC1 constitute the majority of splenic cDC1 in the steady state [6,12,47]. BTLA then engages herpesvirus entry mediator (HVEM) in CD4^+^ T cells to modulate the CD5-dependent resistance of developing pTregs to effector-differentiating cytokines such as IL-4, IL-6, and IFN-γ [47,92,101,103]. Therefore, functions of the BTLA–HVEM–CD5 axis stabilize and promote the process of pTreg conversion [101]. Overall, these mechanisms mediated by BTLA^hi^ cDC1 are crucial for a vigorous pTreg conversion, which is key to the de novo induction of specific peripheral tolerance.

## 4. Other Cell Surface Molecules Used for Antigen Delivery to Dendritic Cells

The successful delivery of antigens via DEC-205 prompted the introduction of recombinant antibodies targeting other surface molecules present on cDC and pDC, and many such antibodies used a similar design originated by the recombinant anti-DEC-205 but utilized V regions from the original antibodies specific for the corresponding molecules [9,45,46,47,55,65,66,74,104,105]. In addition to recombinant chimeric anti-CD11c to deliver antigens to all murine cDC, irrespectively of subsets, multiple other antigen-targeting antibodies have been used. Langerin (CD207) is a transmembrane protein expressed on the cell surface of Langerhans cells as well as on some cDC1 of the spleen and skin, draining lymph nodes in mice, and has been identified on human Langerhans cells and tissue cDC2 [106,107,108,109,110,111,112,113,114,115,116,117,118]. Antigen-delivering anti-Langerin antibodies target antigens to Langerin^+^ cDC of the spleen and peripheral lymph nodes and dermal Langerin^+^ cDC, resulting in lessened EAE symptom severity in a manner similar to that observed following administration of anti-DEC-205-MOG [9,46,74,104]. Trem-like 4 (Treml4) is a member of the “triggering receptor expressed on myeloid cells” family that binds apoptotic or necrotic cells and is expressed in mice, predominantly on cDC1 as well as splenic macrophages [66,119]. The targeting of diverse antigens to Treml4^+^ cDC1 by anti-Treml4 antibodies elicited both CD4^+^ and CD8^+^ T cell responses, but the effects of antigen delivery on disease severity in various models, including tumor transplantation and EAE, remain to be more fully elucidated [46,66]. C-type lectin domain family 9A is an endocytic C-type lectin receptor that binds necrotic cells and is also known as dendritic cell natural killer lectin group receptor-1 (Clec9a, DNGR-1). It is primarily expressed by murine cDC1 and pDC, human cDC1, and some B cells, and antigens taken up in a Clec9a-dependent manner can be presented on MHC-I and MHC-II [120,121,122,123,124,125,126,127]. Under steady state conditions, antigens delivered to cDC using anti-Clec9a chemically conjugated antibodies and presented on MHC-II prompted the differentiation of pTregs [44]. In contrast, antigen targeting Poly(I:C)-matured DC using recombinant anti-Clec9a antibodies resulted in Th1 CD4^+^ and CD8^+^ T cell priming comparable to that observed following administration of anti-DEC-205-Ag or anti-Langerin-Ag [9,74]. Targeting through Clec9a in a humanized mouse system resulted in a powerful priming of CD8^+^ T cells [128].

Currently, fewer options exist for targeting antigens specific to the cDC2 lineage. DC inhibitory receptor 2 lectin (DCIR2) (also known as Clec4a4) is expressed by some murine splenic cDC2, while Clec4a (DCIR), the sole human DCIR family member, is more broadly expressed, including on pDC [45,129,130,131,132,133,134]. Therefore, antigens can be delivered to murine cDC2 using anti-DCIR2, and a targeted antigen delivery through DCIR2 was employed to induce pro-immunogenic immune responses, which resulted in desirable outcomes such as prolonged survival in tumor models [45,56,57,133].

Sialic acid binding Ig-like lectin H (Siglec-H) and bone marrow stromal cell antigen 2 (BST2), which correlate with human pDC-expressed molecules, were successfully used for antigen delivery to murine pDC, resulting in pro-immunogenic or tolerogenic T cell responses [8,55,65,135,136,137,138,139]. In the steady state, antigens delivered to pDCs blocked autoimmune reactions, whereas in the presence of an adjuvant, such antigen targeting may lead to antiviral and anti-tumor responses [55,65].

## 5. Translational Perspectives of Targeted Antigen Delivery to Dendritic Cells

Humanized recombinant antibodies analogous to their murine counterparts described above have been considered as antigen-delivery vehicles in patients. Although the expression of human DEC-205 is not limited to cDC1, human cDC1 highly express DEC-205 [140,141]. The in vivo administration of anti-hDEC-205 antibodies fused to Epstein–Barr virus or human immunodeficiency (HIV) antigens together with the adjuvant Poly(I:C) elicited antiviral T cell responses [142,143,144,145]. Similarly, vaccination with anti-hDEC-205-NY-ESO-1 tumor antigen in conjunction with Toll-like receptor (TLR) agonists elicited an anti-tumor immune response and tumor regression in some patients with multiple advanced malignancies refractory to other therapies [146,147]. Overall, the anti-DEC-205-mediated delivery of antigens from pathogens and tumors is likely to further render these recombinant antibodies as central to the design of new vaccine-based immunotherapies. In addition to full immunoglobulins, single-chain fragment variable region (scFv) and single-domain antibody (sdAb) have also been used for antigen delivery under multiple immune conditions [9,33,59,148,149,150,151,152,153].

In addition to the applications against infections and tumors, the vast body of work obtained from the animal disease models described above suggests the targeted antigen delivery to tolerogenic cDC1 as possible new efficacious strategies for corresponding therapies in patients with autoimmune diseases, such as MS. Despite a substantial progress in developing new immunotherapies against MS, currently available immunotherapy protocols are only partially effective, and most of these approaches are burdened by side effects [30]. Therefore, the new approaches for immunomodulation of MS should specifically remove functions of offending immune cells without impairing immune responses against pathogens and tumors [30]. The neural inflammation responsible for neurological symptoms in MS and its animal disease model EAE results from the infiltration of nervous tissues by perivascular CD4^+^ T that gives rise to the Th1 and Th17-dependent autoimmune process [62,92,154,155]. Immunization with neural antigens, such as MOG, myelin basic protein (MBP), or proteolipid protein (PLP), leads to different variants of EAE that resemble the human progressive and relapsing–remitting forms of MS [92]. Previous research from multiple groups has shown the efficacy of the introduction of MOG, MBP, and PLP in a non-immunogenic context for blocking the subsequent induction of the corresponding forms of EAE [92,156,157]. However, a long-term administration of such antigens without control over which antigen-presenting cells (APCs) present them to T cells in vivo may not guarantee the certainty of the ultimate therapeutic results, and instead could lead to a generation of autoimmune pre-effectors and effectors [30]. Therefore, in addition to focusing on neural-derived antigens that are specifically recognized only by the corresponding encephalitogenic T cells, and thus no other T cells, such specific antigens should be delivered only in a “tolerogenic” context to ensure the specific elimination or functional inactivation of antigen-activated encephalitogenic T cells. These goals might be achieved by approaches that rely on transfers of autologous DC with tolerogenic functions induced in vitro, and similar approaches are now in clinical trials [9,11,30]. However, such approaches are going to require costly treatments with individually prepared DC for each patient while not guaranteeing the functional activity of such transferred DC. Instead, the direct delivery of antigens to cDC with specialized tolerogenic functions in vivo would allow for a better control of the ensuing immunological processes and maximize the de novo induction of antigen-specific pTregs [30,31,32]. In general, Tregs play a crucial role in protection and recovery from EAE and MS by suppressing autoreactive T cells, and functional defects of Tregs have been implicated in the pathogenesis of MS [92]. It is now well established that neuroinflammation can be ameliorated or even completely prevented by antigen-specific pTregs that are induced systemically in the secondary immune organs in response to the relevant neuronal antigens, and confer a long-lasting tolerance that can suppress local effector responses in the CNS and prevent EAE [47,62,92]. Therefore, strategies focused on delivering the neural antigens specifically to tolerogenic cDC, such as BTLA^hi^ cDC1, could offer the most effective means for therapeutically relevant induction of tolerance. Further manipulations of these specific BTLA-mediated mechanisms could open new avenues for immunomodulation by manipulating the specific formation and functions of the relevant pTregs. These approaches allow for highly specific immunomodulation that is focused only on autoreactive encephalitogenic T cells, without impairing other immune responses, and would therefore be more selective and have fewer side effects. Further, such approaches could be extended to other diseases. Overall, antigen delivery through DEC-205 and other cDC surface proteins has allowed for a comprehensive analysis and utilization of the outcomes of T cell activation and priming by specific cDC subsets. Therefore, future applications should carefully consider the specific advantages and disadvantages of delivering antigens to defined cDC subsets with diverse functions.

## Figures and Tables

**Table 1:** Summary of the different DC surface receptors used for delivery of specific antigens in different disease models.

Receptor	Disease (Model)	Antigen(s)	Reference(s)
BST2	Melanoma (B16-OVA)	OVA	[55]
	Vaccinia-OVA Infection	OVA	[55]
DCIR2	Diabetes (non-obese diabetic)	BDC2.5-stimulatory mimetope	[56]
	Melanoma (B16F10-OVA)	OVA	[57]
DEC-205	Arthritis (proteoglycan-induced)	Cartilage proteoglycan	[52]
	Breast cancer (*neu*-expressing NT2.5)	HER2/neu	[58]
	Diabetes (INS-HA/TCR-HA transgenic)	Hemagglutinin (HA)	[54]
	Diabetes (non-obese diabetic)	Islet-specific glucose-6-phosphatase catalytic subunit-related protein	[49]
	Diabetes (non-obese diabetic)	Proinsulin 2	[50]
	Graft-versus-host disease (Skin transplant)	Type XVII collagen	[53]
	Inflammatory Bowel Disease (VILLIN-HA transgenic)	Hemagglutinin (HA)	[51]
	Melanoma (B16F10 and RET)	gp100	[59]
	Melanoma (B16)	Tyrosinase-related protein 2 and gp100	[60]
	Melanoma (B16F10-OVA)	OVA	[57,61]
	Multiple Sclerosis (EAE)	MOG	[36,46,47,62]
	Vaccinia-OVA Infection	OVA	[61]
	*Yersinia pestis* Infection	*Yersinia pestis* LcrV	[63,64]
Langerin	Multiple Sclerosis (EAE)	MOG	[46]
Siglec-H	Multiple Sclerosis (EAE)	MOG	[65]
Treml4	Breast cancer (*neu*-expressing NT2.5)	HER2/neu	[66]

DC: dendritic cells; OVA: ovalbumin; MOG: myelin oligodendrocyte glycoprotein.
